# Tocilizumab Is Associated with Increased Risk of Fungal Infections among Critically Ill Patients with COVID-19 and Acute Renal Failure: An Observational Cohort Study

**DOI:** 10.3390/life13081752

**Published:** 2023-08-16

**Authors:** Barrett J. Burger, Sarenthia M. Epps, Victor M. Cardenas, Rajani Jagana, Nikhil K. Meena, William T. Atchley

**Affiliations:** 1 Division of Pulmonary and Critical Care Medicine, University of Arkansas for Medical Sciences, 4301 West Markham Street, Little Rock, AR 72205, USA; smclelland@uams.edu (S.M.E.); nmeena@uams.edu (N.K.M.);; 2 Department of Epidemiology, University of Arkansas for Medical Sciences, 4301 West Markham Street, Little Rock, AR 72205, USA

**Keywords:** COVID-19, tocilizumab, fungemia, fungal infection, fungal pneumonia, renal replacement therapy

## Abstract

Research Question: Does treatment with tocilizumab increase the risk of a fungal infection in critically ill patients with coronavirus-19? Background: Numerous therapies have been evaluated as possible treatments for coronavirus-2019 caused by severe acute respiratory syndrome coronavirus-2. Tocilizumab is a humanized monoclonal antibody directed against the interleukin-6 receptor that has found a role as a therapy for patients with severe coronavirus-19 pneumonia. The immunomodulatory effects of tocilizumab may have the unintended consequence of predisposing recipients to secondary infections. We sought to assess the risk of invasive fungal disease and the therapeutic impact of tocilizumab on the hospital length of stay, duration of mechanical ventilation, and intensive-care-unit length of stay in critically ill patients with severe coronavirus-19 pneumonia. Methods: Records of critically ill patients with coronavirus-2019 admitted from March to September 2020 at our institution were reviewed. The risk for fungal infections, intensive-care-unit length of stay, hospital length of stay, and duration of mechanical ventilation in those that received tocilizumab in addition to standard coronavirus-2019 treatments was assessed. Results: Fifty-six critically ill patients treated with dexamethasone and remdesivir for coronavirus-2019 were included, of which 16 patients also received tocilizumab. The majority of the cohort was African American, Asian, or of other ethnic minorities (53.6%). Invasive fungal infections occurred in 10.7% of all patients, and infection rates were significantly higher in the tocilizumab group than in the control group (31.2% vs. 2.5%, risk difference [RD] = 28.8%, *p* < 0.01). The increased risk in the tocilizumab group was strongly associated with renal replacement therapy. There was a dose–response relationship between the risk of fungal infection and number of tocilizumab doses received, with 2.5% of infections occurring with zero doses, 20% with a single dose (RD = 17.5%), and 50% with two doses (RD = 47.5%) (trend test *p* < 0.001). In addition, ICU LOS (23.4 days vs. 9.0 days, *p* < 0.01), the duration of mechanical ventilation (18.9 vs. 3.5 days, *p* = 0.01), and hospital length of stay (LOS) (29.1 vs. 15.5, *p* < 0.01) were increased in patients that received tocilizumab. Conclusions: Repurposed immunomodulator therapies, such as tocilizumab, are now recommended treatments for severe coronavirus-2019 pneumonia, but safety concerns remain. In this early pandemic cohort, the addition of tocilizumab to dexamethasone was associated with an increased risk of fungal infection in those that were critically ill and received renal replacement therapy. Tocilizumab use was also associated with increased ICU and hospital LOSs and duration of mechanical ventilation.

## 1. Background

Several medications have been repurposed for the treatment of coronavirus-2019 (COVID-19) caused by the severe acute respiratory syndrome coronavirus-2 (SARS-CoV-2) [1]. Many of these treatments have immunomodulatory effects and, therefore, may predispose patients to secondary infections. Tocilizumab is a humanized monoclonal antibody directed against the interleukin-6 (IL-6) receptor, approved for the treatment of rheumatoid arthritis, giant cell arteritis, Castleman disease, and cytokine release syndrome [2,3]. Tocilizumab was originally proposed as a potential therapy for COVID-19 after markedly elevated serum IL-6 levels were reported in early cases from Wuhan and was associated with reduced survival [4,5,6]. Toniati and colleagues reported a series of 100 COVID-19 patients treated with tocilizumab that showed a rapid improvement in respiratory status after receiving tocilizumab [7]. Likewise, Sciascia and colleagues reported a series of patients that showed improvements in their respiratory parameters when tocilizumab was given within six days of admission [8]. A series of patients treated with tocilizumab at Yale also had lower inflammatory markers as well as higher-than-expected survival, and multivariable analyses from a retrospective case series found that tocilizumab may be associated with reduced risks of mechanical ventilation and death [9,10]. Likewise, case series have investigated the combined use of tocilizumab with dexamethasone, and at least one report showed survival benefit when compared with dexamethasone alone [11].

More recently, the COVACTA trial failed to demonstrate improved clinical status or decreased mortality when compared to placebo at 28 days in a cohort of patients hospitalized with severe COVID-19 [12]. A cohort of 154 ventilated patients reported by Somers et al. had improved mortality but an increased risk of superinfection (largely methicillin-resistant *Staphylococcus aureus*) [13]. Concomitantly, the REMAP-CAP published promising data collected from their domain of critically ill patients receiving significant oxygen support and tocilizumab. These patients experienced a greater number of cardiovascular- and respiratory-support–free days when compared with placebo [14]. These data contributed to the European Society of Clinical Microbiology and Infectious Diseases approving tocilizumab for the treatment of severe COVID-19 with a strong recommendation [15,16].

We observed a relatively high incidence of secondary fungal infections among critically ill patients with COVID-19 at our institution and performed a retrospective study to assess whether tocilizumab treatment was associated with an increased risk for fungal infection.

## 2. Methods

We retrospectively extracted the medical records of patients with COVID-19 admitted to the intensive care unit (ICU) at our institution from March to September 2020. The period of data collection was chosen to begin when ICU patients uniformly received dexamethasone, which is a National Institutes of Health (NIH) Grade IIb guideline-based treatment for COVID-19 [17]. All data were collated and managed using the REDCap (Research Electronic Data Capture) electronic data capture tool hosted at UAMS [18].

Adults ≥18 years of age admitted to the ICU during their hospitalization with polymerase chain reaction–confirmed SARS-CoV-2 infection were identified (*n* = 128) and included in the study if they received at least 5 doses of remdesivir (≥100 mg/dose) and dexamethasone (≥6 mg/dose). At the time of data collection, tocilizumab was still under investigation as a treatment for severe COVID-19 and was only considered for use in patients admitted to the intensive care unit with increasing supplemental oxygen requirements despite dexamethasone. We compared important outcomes for those who received tocilizumab to those who did not (control), including ICU and hospital lengths of stay (LOS), the duration of mechanical ventilation (MV), and the risk of death during the hospitalization. The study was approved by the Institutional Review Board of UAMS (IRB #260970).

Patients were excluded if they had a history of a fungal infection, received any COVID-19-specific therapy before admission to our hospital, or received other immunomodulatory treatments (e.g., chemotherapy, disease-modifying antirheumatic drugs) within seven days of admission. Fungal infection was defined as either culture from a sterile site (i.e., blood), identification on molecular assays (e.g., aspergillus galactomannan antigen), or culture from bronchoalveolar lavage that was clinically considered pathogenic and treated. Cultures of fungal species from tracheal aspirates, sputum, or urine were not considered pathogenic.

## 3. Baseline Variables

Clinically relevant data, including patient demographics, the duration of hospitalization, respiratory support requirements (e.g., oxygen flow rate), medical comorbidities, medication administration records, pertinent laboratory data (e.g., culture results), and vital status at hospital discharge, were collected.

## 4. Statistical Analysis

Data analyses were performed using SAS Software for Windows, version 9.4 (SAS Institute Inc., Cary, NC, USA). A *p*-value < 0.05 was considered significant for all tests. Categorical variables were expressed as mean with standard deviation or median with 25th–75th percentile ranges. We tested the null hypothesis of no difference in the frequency of fungal infections between the tocilizumab and the control groups (risk difference [RD] = 0) using a Fisher exact test. A dose–response analysis was performed testing the null hypothesis of no trend in the risk of developing a fungal infection by the number of doses (none, 1, or 2+) of tocilizumab using the Cochran–Armitage chi-square test for a linear trend. For secondary outcomes, ICU LOS, hospital LOS, and duration of MV were compared using *t*-tests and regression analysis. Results were visualized using histograms and smoothed using Kernel density estimations to view the most frequent outcomes. Outlying results were visualized using box plots. Results for the risk of fungal infection were stratified by age, sex, race, APACHE II score, and continuous renal replacement therapy (CRRT). The assessment of interaction was presented using the additive scale [19]. Adjusted estimates were obtained using a log-binomial regression (with the identity option in the model statement) to estimate the RD [20].

## 5. Results

### 5.1. Demographic and Clinical Characteristics

A total of 56 patients received both dexamethasone and remdesivir for severe COVID-19 and required ICU admission. Sixteen of these patients (tocilizumab group) also received at least one dose of tocilizumab (≥8 mg/kg/dose). Baseline demographics, clinical characteristics, and pertinent laboratory values of tocilizumab and control groups are displayed in Table 1. Subjects in the groups were similar with regard to demographics, which consisted of a relatively large African American (35.7%) and Hispanic population (17.9%). Slightly more Hispanic patients received tocilizumab (37.5% of the tocilizumab group vs. 10% of the control group). A higher percentage of patients in the tocilizumab group also received CRRT (37.5%).

### 5.2. Clinical Outcomes

Fungal infection was identified in 10.7% (6/56) of all patients, which uniformly reflected candidemia (*Candida albicans* on venous blood cultures). Candidemia rates were significantly higher in the tocilizumab group (5/16, 31.2%) than in the control group (1/40, 2.5%, *p* < 0.01), resulting in an RD of 28.8% (95% confidence interval (CI) = 5.5–52.0, *p* < 0.01). There was a significant dose–response relationship for tocilizumab, with fungal infections occurring in 2.5% (1/40) of patients in the control group, 20% (2/10) of patients that received one dose of tocilizumab (RD = 17.5), and 50% (3/6) of patients receiving two doses (RD = 47.5). The Cochran–Armitage trend test rejected the null hypothesis of no linear trend in the proportion of patients with fungal infection versus the increased number of tocilizumab doses (*p* < 0.001).

Age, gender, race, disease severity (APACHE II > 20), or indwelling central catheter did not alter the effect of tocilizumab on fungal infections, but the use of CRRT did confer excess risk (Table 2). The effect of tocilizumab and CRRT alone was null, while the effect of tocilizumab was very strong in the subset of patients on CRRT, suggesting an additive effect. The joint effect of tocilizumab and CRRT on RD, adjusted for gender, race/ethnicity, the presence of a central line, and disease severity, was 77.2% (95% CI = 15.7–100.0, *p* = 0.04). No other factor was statistically significant after adjustment for the above covariates.

**Figure 1:**
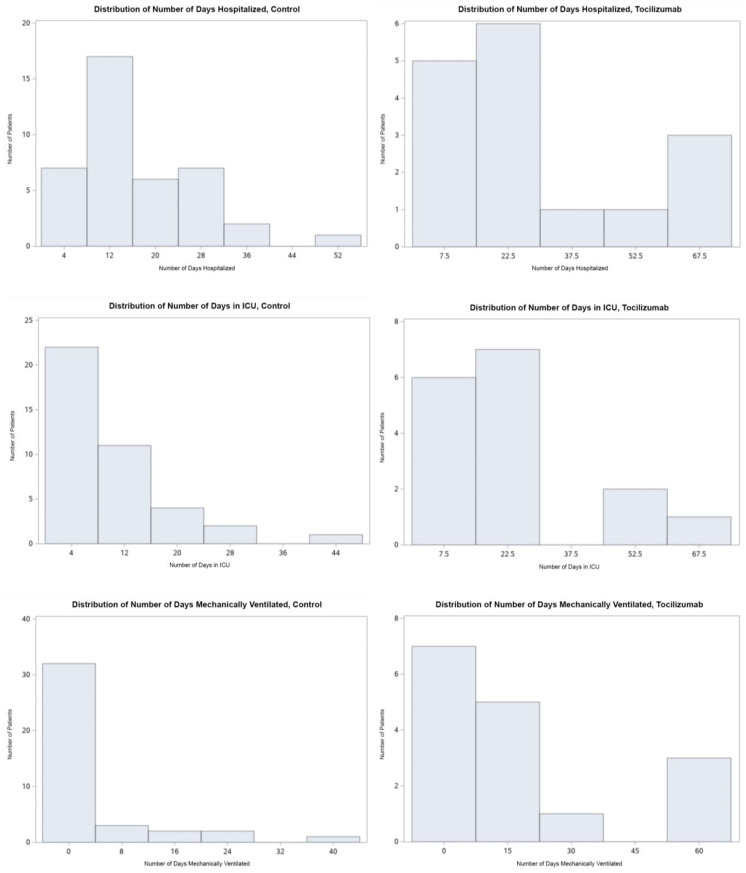
(Top to bottom, L to R) Numerical distribution of number of days hospitalized for patients who did not receive tocilizumab and those who did, number of days in the ICU for patients who did not receive tocilizumab and those who did, and number of days mechanically ventilated for patients who did not receive tocilizumab and those who did.

**Figure 2:**
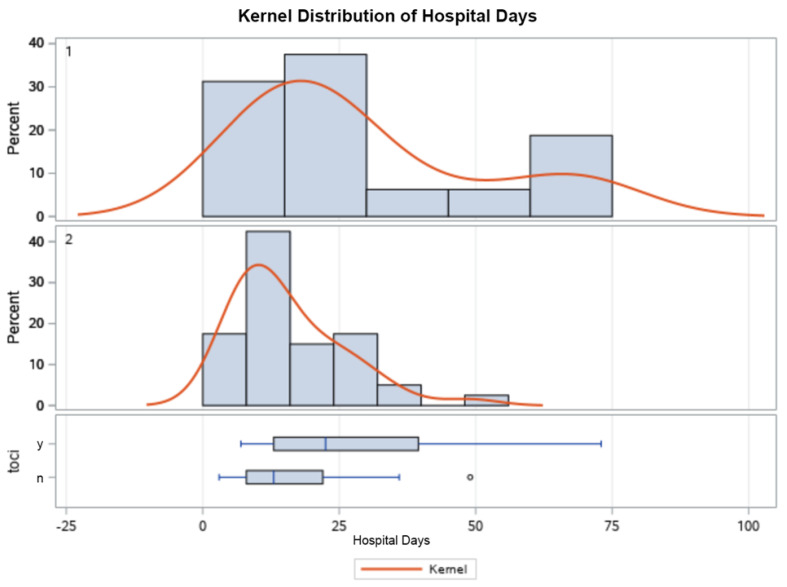
Kernel population density distribution of mean hospital days in patients who received tocilizumab (**top**), those who did not (**middle**), and box plot (**bottom**) showing the IQR of those who did (y) and did not (n) receive tocilizumab.

**Figure 3:**
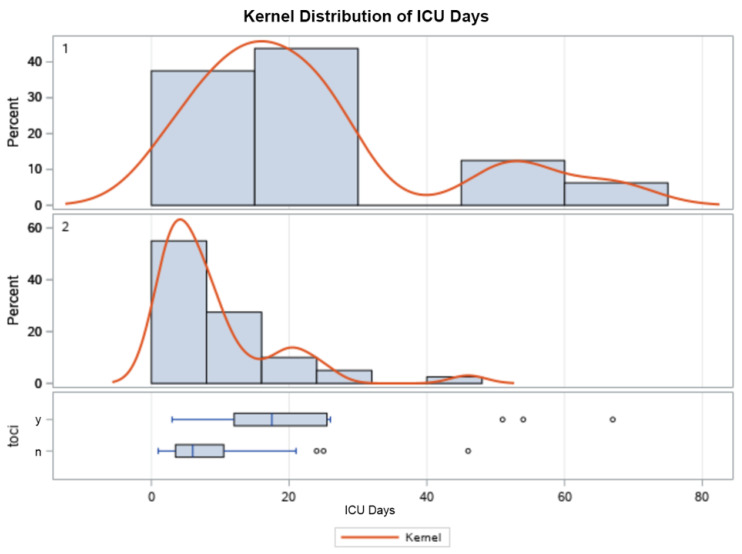
Kernel population density distribution of mean ICU days in patients who received tocilizumab (**top**), those who did not (**middle**), and box plot (**bottom**) showing the IQR of those who did (y) and did not (n) receive tocilizumab.

**Figure 4:**
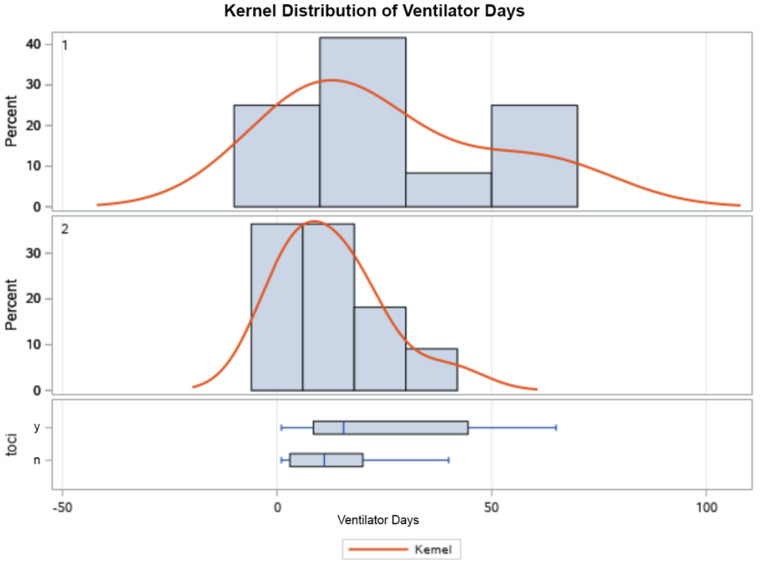
Kernel population density distribution of mean ventilator days in patients who received tocilizumab (**top**), those who did not (**middle**), and box plot (**bottom**) showing the IQR of those who did (y) and did not (n) receive tocilizumab.

As outlined in Table 3, the mean ICU LOS, hospital LOS, and length of MV were significantly higher in those that received tocilizumab (Figure 1, Figure 2, Figure 3 and Figure 4). Additionally, the ICU LOS (37.3 vs. 19.1 days) and hospital LOS (38.3 vs. 25.8 days) were both significantly prolonged in patients that developed a fungal infection (both ICU and hospital LOS *p* < 0.01). The mean duration of MV was likewise increased in those who developed a fungal infection (35.8 days) versus 19.5 days in those who did not (*p* < 0.01).

Finally, there were more hospital deaths among those who received tocilizumab (43.8%) than controls (27.5%) (RR = 1.6, CI = 0.8–3.4). There was a trend toward increased hospital deaths among cases of fungal infection (50.0%) compared to non-cases (30.0%), but the RR (1.7) was not significantly different from the null (95% CI = 0.7–41.4)

## 6. Interpretation

We sought to determine whether the addition of tocilizumab to steroid- and remdesivir-based treatment regimens for severe COVID-19 pneumonia requiring ICU admission increased the risk of developing fungal infections and likewise altered important ICU outcomes. The cohort included a relatively high percentage of African American, Asian, and Hispanic patients, as well as a high number of patients with diabetes mellitus, consistent with reports that diabetes is associated with increased severity of COVID-19 [21].

We found the risk of a patient incurring a fungal infection was significantly higher in the group that received tocilizumab with a strong dose–response relationship wherein the risk of infection increased in concert with the number of doses of tocilizumab received. However, the risk was modified by concurrent renal failure as the tocilizumab-treated patients who required CRRT carried much of the excess risk of fungal infection. We attempted to control for other factors associated with fungal infections in critically ill patients and found that tocilizumab, in conjunction with CRRT, remained independently associated with increased risk.

The tocilizumab group trended toward a lower median APACHE II score at ICU admission, suggesting a lower severity of illness. However, other indices of disease severity, including the use of mechanical ventilation for respiratory support and the need for renal replacement therapy, trended higher in the tocilizumab group. To account for some of the differences in the severity of illness between groups, the risk of fungal infection in each group was adjusted for age, gender, race, APACHE II score, and central catheter placement, and only tocilizumab treatment was associated with an increased risk for invasive fungal disease.

When examining the secondary outcomes and the distribution of ICU days, ventilator days, and hospital days between groups, all were skewed heavily to the R of the *x*-axis, as expected with the natural course of illness. Results were appropriately log-transformed, and fungal infections caused significant lengthening of each of these outcomes when compared using t-tests for each variable. Kernel analysis also showed a greater density of patients in the tocilizumab group having lengthening of each outcome. Receiving tocilizumab and incurring a fungal infection increased the number of ICU days, ventilator days, and hospital days significantly and might also increase case fatality.

## 7. Discussion

Clinical trials of tocilizumab for COVID-19 have reported mixed efficacy depending on the setting and expediency with which the medication is administered, while the question of relative risk for secondary infections when using tocilizumab in this setting remains largely unanswered [22,23]. For example, a randomized, placebo-controlled trial testing tocilizumab for the treatment of COVID-19 outside of the ICU did not show a significant reduction in the World Health Organization-Clinical Progression Score (WHO-CPS), but there was a possible reduction in the risk of MV or death by day 14 [24,25]. This trial is in congruence with Gupta et al., who reported an association with lower in-hospital mortality in patients with COVID-19 who received tocilizumab in the first 2 days of their admission to the ICU [26]. In contrast, for non-ICU patients, a recent randomized trial showed no clear benefit in disease progression with tocilizumab [27]. Further muddying the waters, Stone et al. determined that tocilizumab did not prevent intubation or death in moderately ill hospitalized patients, while a Brazilian study was halted early because of increased deaths in the tocilizumab arm of a randomized trial [28,29]. More recently, the REMAP-CAP group reported a reduction in the RR of death by 24% with tocilizumab or sarilumab, with the majority of clinical benefit noted when the medication was administered within 72 h of presentation [14]. Despite the heterogeneous outcomes reported above, the NIH now recommends immunomodulators such as tocilizumab for severe COVID-19 pneumonia as adjunct treatment for patients requiring high/escalating oxygen support and/or rising inflammatory markers despite dexamethasone.

Many of the above-referenced randomized trials assessing the efficacy of tocilizumab in COVID-19 did not require patients to receive dexamethasone or an equivalent dose of corticosteroid, which is now standard of care for the treatment of severe COVID-19. This may account for some of the differences in efficacy and leaves the question of infection risk largely unanswered for cases of COVID-19 treated with a combination of corticosteroid and IL-6 blockade. As tocilizumab now has established use in critically ill patients with COVID-19, the combined immunomodulatory effects of corticosteroid with tocilizumab and the risk for opportunistic infections in the critically ill population warrant further study. Antinori and colleagues first cautioned the medical community about the possibility of increasing the risk of candidemia with the widespread use of tocilizumab in a letter to the editor of Autoimmunity Reviews in July 2020, noting the risk in literature reviews [2,30].

To our knowledge, no previous study has identified an increased risk for fungal infection with tocilizumab treatment in patients with severe COVID-19 and renal failure. Prolonged hospitalizations due to persistent respiratory failure are unfortunately common in severe COVID-19 pneumonia, and there appears to be an increased risk for acute kidney injury (AKI), with at least one study reporting 20–40% of patients hospitalized with COVID-19 will develop AKI. Furthermore, in patients hospitalized with COVID-19, kidney injury itself appears to be associated with an increased risk of serious bacterial and fungal infections [31,32]. In other studies assessing tocilizumab, Gupta and colleagues reported numerically more infections, and Viega et al. reported no difference in secondary infection between groups, but no trial has looked specifically at the effects of renal replacement therapy and tocilizumab [29,33]. Our study demonstrates an almost additive effect from tocilizumab and kidney injury on the risk for fungal infection in severe COVID-19, where there is already a significant risk of candidemia in these critically ill patients.

## 8. Limitations

This study has limitations owing to its observational nature and single-institution design, although other institutions have reported similar findings [31,34]. The exclusion of all potential confounders is virtually impossible, and biases regarding the individual use of tocilizumab and the timing and number of doses utilized cannot be completely excluded. While the group that received tocilizumab did have lower APACHE II scores at admission, the increased utilization of mechanical ventilation and renal replacement therapy in the treatment group indicates a likely bias toward tocilizumab use in patients who were more ill during their hospital course. Our data collection systems were unable to delineate the number of days or dose of dexamethasone used or if there were additional steroid formulations administered, which could impact the development of secondary infections. The total case number and absolute number of fungal infections in this cohort are relatively small, and our risk estimates have correspondingly wide confidence intervals. Thus, the strong association between tocilizumab treatment and invasive fungal infections we found warrants continued study in larger COVID-19 registries or clinical trials enrolling critically ill patients. Finally, we did not address whether other co-infections could have been present or incited by tocilizumab administration.

## 9. Conclusions

The current evidence, including randomized clinical trials, largely demonstrates the significant benefits of the use of tocilizumab in critically ill patients with severe COVID-19 pneumonia, but the subgroups of these patients who may be harmed by the development of secondary infections are not clear. In this cohort of critically ill patients with severe COVID-19 treated with dexamethasone and remdesivir, we found a significant risk of developing secondary fungal infections in patients with renal failure who received treatment with tocilizumab. Thus, patients with severe COVID-19 critical illness requiring the use of combined immunomodulatory treatments in the setting of AKI may represent a particularly vulnerable patient population with regard to the risk of secondary fungal infections, warranting further study. These secondary fungal infections likewise greatly impacted morbidity, as suggested by the longer ICU and hospital LOSs and duration of MV for those who received tocilizumab and developed fungal infections. Future studies are warranted from larger patient cohorts and randomized trials to assess for this risk, with special attention paid to the addition of IL-6 inhibitors to corticosteroid therapies.

## Figures and Tables

**Table 1:** APACHE II scoring reflects those that had requisite data collected on admission to ICU to be calculated. IQR = interquartile range, 3rd quartile minus 1st quartile (Q_3_–Q_1_). A total of 37 patients had requisite data available for APACHEII scoring.

	All Patients (n = 56)	Control (n = 40)	Tocilizumab (n = 16)
Demographics			
Gender, n (%)			
Female, n (%)	27 (48.2)	21 (52.5)	6 (37.5)
Age, median (IQR)	60 (22.0)	60 (24.5)	58 (19.5)
Race, n (%)			
Black, n (%)	20 (35.7)	14 (35.0)	6 (37.5)
White, n (%)	23 (41.1)	19 (47.5)	4 (25.0)
Hispanic, n (%)	10 (17.9)	4 (10.0)	6 (37.5)
Other, n (%)	3 (5.3)	3 (7.5)	0 (0)
Comorbid disease, n (%)			
Diabetes, n (%)	25 (44.6)	17 (42.5)	8 (50)
Chronic liver disease, n (%)	1 (1.8)	1 (2.5)	0 (0)
Renal failure, requiring replacement therapy, n (%)	10 (17.9)	4 (10.0)	6 (37.5)
Laboratory results, maximum value while in ICU, median (IQR)			
AST U/L (IQR)	44 (62)	48.5 (63)	42.5 (67)
ALT U/L (IQR)	47 (70)	45 (66)	48 (82)
Total bilirubin mg/dL (IQR)	0.9 (0.7)	0.9 (0.7)	1.1 (0.8)
Received TPN while inpatient, n (%)	1 (1.9)	1 (2.5)	0 (0)
Indwelling central catheter, n (%)	26 (65)	15 (37.5)	11 (73.3)
APACHE II score > 20, n (%)	7 (12.5)	3 (15.0)	4 (28.6)
APACHE II score (IQR)	14 (9)	15 (9)	12.5 (8)

**Table 2:** Modification of the effect of tocilizumab use on the risk of fungal infection by CRRT in COVID-19 patients. RD = risk difference. Excess risk due to interaction = 83.3%–0.0%–0.0% + 2.8% = 86.1% > 0, indicating super additivity. The adjusted RD for those who were both on tocilizumab and CRRT was 77.2 (95% CI = 15.7, 100.0%), adjusting for age, gender, race/ethnicity, and disease severity.

Continuous Renal Replacement Therapy (CRRT)	Tocilizumab				RDs (95% CI) for Tocilizumab within CRRT Strata
	No		Yes		
	Fungal Infection	Patients	Fungal Infections	Patients	
No	1	36	0	9	−2.8 (−8.1, 2.6)
RD (95% CI)	0		−2.8 (−8.1, 2.6)		*p* = 1.0
	(Referent)		*p* = 1.0		
Yes	0	4	5	6	83.3 (53.5, 113.2)
RD (95% CI)	−2.8 (−8.1, 2.6)		80.6 (50.3, 100.0)		*p* < 0.01
	*p* = 1.0		*p* < 0.01		

**Table 3:** Relevant ICU outcomes. IQR = interquartile range, 3rd quartile (Q_3–_Q_1_).

Characteristic	All Patients (n = 56)	Control (n = 40)	Tocilizumab (n = 16)
Hospital LOS, median days (IQR)	14 (15.5)	13 (14)	22.5 (26)
ICU LOS, median days (IQR)	8 (14.5)	6 (7)	17.5 (16)
Mechanically ventilated, n (%)	23 (41.1)	11 (27.5)	12 (75)
Duration of MV ^1^, median days (IQR)	14 (16)	11 (17)	15.5 (35)
Death ^2^, n (%)	18 (32)	11 (27.5)	7 (43.7)

^1^ Only patients who were mechanically ventilated were used in the calculation for the number of days mechanically ventilated. ^2^ Death refers to in-hospital mortality.

## Data Availability

Deidentified datasets used during this study are available from the corresponding author via reasonable request.

## Abbreviations

COVID-19	coronavirus-2019
SARS-CoV-2	severe acute respiratory syndrome coronavirus-2
IL-6	interleukin-6
ICU	intensive care unit
NIH	National Institutes of Health
MV	mechanical ventilation
LOS	length of stay
CRRT	continuous renal replacement therapy
RD	risk difference
WHO-CPS	World Health Organization-Combined Positive Score
